# The Role of Psychological Capital and Work Engagement in Enhancing Construction Workers' Safety Behavior

**DOI:** 10.3389/fpubh.2022.810145

**Published:** 2022-03-04

**Authors:** Muhammad Shoaib Saleem, Ahmad Shahrul Nizam Isha, Yuzana Mohd Yusop, Maheen Iqbal Awan, Gehad Mohammed Ahmed Naji

**Affiliations:** ^1^Department of Management and Humanities, Universiti Teknologi PETRONAS, Seri Iskandar, Malaysia; ^2^Faculty of Medicine, Universiti Sultan Zainal Abidin, Kuala Terengganu, Malaysia

**Keywords:** psychological capital, work engagement, safety performance, safety compliance, safety participation, construction industry

## Abstract

**Objectives:**

Construction is one of the unsafe industrial sectors, causing a considerable amount of harm to its workforce and organizations globally. Only a handful of research evidence has been found evaluating individuals' cognitive and engagement-related constructs to improve occupational safety. Psychological Capital (PsyCap) can have a promising impact on construction workers' psychological health, possibly leading to positive performance. Limited studies have tested PsyCap and work engagement regarding safety specifically in the context of the construction industry, with non-harmonious findings.

**Methods:**

The proposed framework was assessed through the structural equation modeling (SEM) technique along with bootstrapping for mediation analysis. Responses were collected from different states of Malaysia from 345 construction workers. PsyCap dimensions (hope, efficacy, resilience, and optimism) were measured in connection with safety compliance and safety participation, with the mediating role of work engagement.

**Results:**

According to findings, hope, optimism, and work engagement have a positive and significant impact on safety compliance. Also, hope, self-efficacy, resilience, optimism, and work engagement have a positive and significant impact on safety participation. Further, self-efficacy and optimism both have a positive impact on work engagement.

**Conclusions:**

PsyCap can be a possible predictor for work engagement, which may enhance safety-related behavior. PsyCap should be treated as a multidimensional instrument to enhance occupational safety. In-depth deliberation is needed by the organization while applying PsyCap to enhance employees' work engagement as well as safety behavior. Practical interventions based on interactive training are proposed to enhance construction industry safety. Other industries can also adapt suitable dimension(s) of PsyCap to safety behavior improvements.

## Introduction

Occupational health and safety issues are an utmost concern for every industry in today's complex and demanding work environments. Out of many industries, the construction industry is the one that has witnessed occupational accidents and injuries at large (1, 2). According to the US Bureau of Labor Statistics (3), around 1,102 fatalities were recorded in the construction industry, only in the year 2019 with an injury rate of 2.8 cases per 100 full-time workers, which is quite worrying. Specifically talking about the Malaysian construction industry, unfortunately, there were around 616 deaths reported from the year 2015 to 2020 only out of 2,000+ officially reported cases, which is a point of great concern for this sector (4). The occupational health condition in Malaysia's construction industry is not exceptional in contrast to worldwide occupational accident data, since this sector is reaching high heights even globally (5–7). The surge in occupational accidents in the construction industry stimulates researchers and academics to offer and evaluate new views, with a particular focus on employee safety behavior. According to the literature, human behavior is responsible for around 80% of occupational accidents, making it the most prevalent causal factor (8–10).

Certain physical, organizational, and psychological aspects may impact the cognitive mechanism behind an employee's safety behavior, although human behavior is unpredictable and may be influenced by both internal and external stimuli (11). Out of many organizational concepts, positive organizational behavior introduced the concept known as PsyCap which is widely utilized in the safety research discipline (12–16). The concept of PsyCap has been widely utilized in connection with employees' behavior, positive performance and satisfaction (17, 18), work systems & performance (19), wellbeing, and learning climate (20), job satisfaction, and organizational citizenship behavior (21) and employee attitude (22).

PsyCap is used as multidimensional a variable in some of the past studies (23, 24). Only a few of the researchers have empirically tested PsyCap through its sub-dimensions regarding safety behavior in the different cultural contexts for the construction industry (15). There prevails incongruities in prior researches and allowed us to explore PsyCap in the Malaysian construction industry. Further, the Malaysian construction workforce faces an environment that is riskier and contains performance pressure and psychological strain, which may negatively govern the psychological conditions of construction workers (25). Consequently, it would be useful to assess if the PsyCap of employees will modify their safety behavior and how the intervention of PsyCap can enhance safety performance overall. More empirical evidence will add value to the utility of PsyCap and its sub-dimensions to predict the behavioral phenomenon.

Referring to the possible outcomes of PsyCap (26), According to the literature, PsyCap, a person-oriented component, is necessary to enhance employee job engagement. Workers with higher levels of Psycap (hope, self-efficacy, resilience, and optimism) are more engaged and productive at work, leading to positive organizational behavior and job satisfaction. Luthans et al. (17), so engaged behavior is expected more often. Some of the studies in past have examined the predicting role of PsyCap for work engagement in different contexts alongside diverse variables i.e., leader and followers' Psycap (27), authentic leadership, fellowship (26), organizational socialization, and leader's PsyCap (28), self-leadership, mindfulness, and PsyCap (29).

Another individual aspect to look upon safety is the involvement and engagement of the workforce deployed. Boeldt (30) stated that an engaged workforce is a safe workforce, emphasizing the importance of an engaged workforce at the workplace. Prior literature also upholds that organizations that surpassed the performance standards are driven by their engaged workforce (31). Engagement at work is defined by having characteristics like vigor, dedication, and absorption (32) and continuous demonstration of them by employees. Before this, work engagement has been associated with variables like Training perceptions (33), engagement and performance (34), daily job demands and fatigue (35), proactive work behavior (31), and job demands, job resources, & burnout (32).

To specify the motivation behind this research, we would like to highlight the following. Using the meta-analytic approach for the construction industry, Xia et al. (36) highlighted the future theoretical avenues under the job strain domain, shifting the conventional wellbeing phenomenon toward the employee's flourishing state of work engagement and a work-related sense of wellbeing that needs to be explored further. The stem of the researchers also presented the conceptual model, representing work engagement as the possible predictor for safety behavior, which yet requires empirical testing in the construction industry (37). However, the quantitative evidence to predict the safety behavior through the work engagement as mediator is still non-existent and allowed us to explore this phenomenon in the Malaysian context. Further, the impact mechanism of work engagement and PsyCap in connection with safety behavior remains to be explored from the multidimensional perspective. To bridge this gap, our study is intended to assess the impact of sub-dimensions of PsyCap on the safety behavior of employees working in the construction industry. Moreover, the mediating role of work engagement will also be assessed, which in harmony with PsyCap may collectively enhance the safety performance of the construction industry. One of the first contributions of this study toward the body of knowledge would be to better understand the predicting role of PsyCap's sub-dimensions for work engagement and ultimately the safety behavior of employees, specifically in the construction industry. Secondly, this study will also shed some light on the multidimensional perspective between PsyCap and safety-related outcomes i.e., highlighting the importance of each dimension of PsyCap independently. Lastly, it will uncover the mediating mechanism of work engagement between PsyCap and safety performance. Our findings may lead to the better operationalization of psychological and behavioral mechanisms to strengthen safety performance for organizations.

## Literature Review

### Psychological Capital

Psychological capital is defined as an individualistic state or aptitude that an individual develops during his/her development and growth. According to some researchers, individuals' PsyCap can be measured, enhanced, and leveraged for better performance outcomes (17). PsyCap is comprised of three different perspectives. Posited by Letcher (38), for the first perspective, an individual's characteristics are the outcome of the interaction between the environment and the personality inheritance from his/her ancestors. This capacity is further elaborated through the five-factor model comprised of, neuroticism, extraversion, openness, agreeableness, and conscientiousness of an individual. The second perspective states that PsyCap is a state that can be utilized to foresee and enhance individual performance (17, 24, 39). The last perspective states that PsyCap is comprised of both individual psychological abilities and characteristics, and its augmentations are possible through interventions of other measures and are relatively stable (40). It is not surprising to have differences in the different dimensions of PsyCap ranging from two to five (23, 24, 41–43). Out of all sub-dimensions of PsyCap, Luthans et al. (44) emphasized four elements i.e., hope, efficacy, resilience, and optimism which are commonly referred to as HERO.

To elaborate on each dimension further; Hope, is an individual's motivation to attain the desired goal, and in the case of non-attainment of the desired goal, hopeful individuals tend to find new and different ways to achieve them (17, 45). Elements like clear goal setting, active participation, advanced preparation, practicing flexibility, cognitive exercises and realignment of goals can increase individual hope (46). The second dimension is Self-efficacy, a personal conviction that an individual possesses to achieve the desired outcomes. Efficacious individuals know their resources, where this self-belief comes from prior experiences, individual mastery, knowledge acquisition, constructive feedback, and psychological reinforcement. Self-efficacious individuals tend to know how to use their psychological and motivational resources for goal attainment which is the outcome of /her social beliefs, prior experiences, learning, and feedback from others (17, 47).

The third dimension is Resilience, referred to as the ability of an individual to stay strong and ambitious in adverse scenarios, and even after seeing any failure or unforeseen and abrupt situations (17). Persistent and tenacious individuals can easily catch up with swiftly changing environments, and they are not only able to recoup from failures, but they have the tendency to absorb criticism and excerpt key learnings (39, 47). Further, the literature states that the resilience of an individual can be augmented through prior evaluation of the associated risks, resources at hand, and a deep understanding of the processes.

The last dimension of PsyCap is Optimism, which is commonly known as an individual's ability to attribute positivity to the ongoing. Optimistic individuals do not indulge themselves with their past for a prolonged period, rather they work on making the present moment more productive, actively seek better alternatives, hold practical views, and maintain flexibility in their thoughts (47). According to Luthans et al. (39) optimistic individuals hold an explanatory style, as they tend to explain situations themselves internally. An optimistic individual is more inclined toward the assessment of causes and clues to ascertain positive emotions and due to the excessive emphasis on analysis and judgments, the optimistic individual is expected to make better choices (17).

### Work Engagement

According to Schaufeli et al. (48), work engagement is an effective, motivational work-related state of an employee, which is made up of characteristics like vigor, dedication, and absorption. Engaged employees have higher degrees of energy, are passionate about their work, and are mostly found deeply submerged into work that their time flies by unnoticeably (49, 50). One of the qualities of engaging workers is that they enjoy challenges and show strong mental resilience, the ability to face challenges while enjoying as well as deep indulgence in their work. Prior literature from the healthcare sector highlighted that, the medical staff was able to take good care and patients were more satisfied when they encounter engaged employees, which not only boosted their performance but overall work effectiveness and quality of care was improved (31).

Work engagement also results in improved interpersonal relationships amongst employees, which in turn fosters a work environment. Together with improved interpersonal relations, work engagement is expected to foster a proactive attitude amongst employees which will ultimately lead to better organizational performance. Macey and Schneider (49) differentiated work engagement into three subcategories i.e., trait engagement (positive view of life and work), state engagement (feeling of energy absorption and effectiveness), and behavioral engagement (extra-role behavior). To relate work engagement with safety performance we will be opting for state and behavioral engagement perspective, as safety performance is comprised of safety compliance (in role behavior) and safety participation (extra-role behavior). The reason why work engagement is of such interest to our study is that it is not just a matter of simple satisfaction with work or at work, loyalty to company or employer, but it is way beyond, as the employees who are engaged are passionate and so committed that they almost invest themselves to help the organization succeed.

Another interesting fact about work engagement is that it fosters happiness and work enjoyableness, where it is not an external reward, but employees tend to work more toward their internal satisfaction by looking at the tasks positively even when they are expected to face strain (51). One of the most prominent reasons to link work engagement with safety behavior is the promising outcome of organizational citizenship behavior (52), which in turn will enhance organizational effectiveness.

### Safety Behavior

Safety behavior is the outcome of actions initiated by employees when they encounter any safety-related situation in an organization. In other words, we can say that safety behavior is the actual safety performance through employees which takes place in the occupational settings (53). Historically, safety performance was assessed via lagging indicators (past incidents/accidents), injury rate, mortality rate which had some shortcomings (54–59). Christian et al. (60) highlighted that relying too much on lagging indicators is expected to produce biased outcomes for the organization as it uses past data. More importantly, lagging indicators do not provide prompt or warnings for safety incidents. Griffin and Neal (61) described safety performance as the work-related behavior of an employee which is related to organizational safety. Griffin and Neal identified two sub-dimensions of safety behavior known as safety compliance and safety participation, which are quite similar to general job-related performance.

Safety Compliance is the in-role behavior, which is supposedly required by employers when they encounter and safety-related situations. It is comprised of those mandatory or required actions which are enforced through policy or part of standard operating procedure. Precise actions which come under safety compliance are, following safety policy, wearing personal protective equipment (PPEs), listening to what the organization says about safety at the workplace. In parallel to this concept, Safety Participation goes beyond safety compliance, where the involvement of employees to participate in safety is voluntary and self-initiated (61). It is not embedded in their roles and neither its part of their responsibility officially, yet they tend to participate in such efforts, which in turn overall facilitates the safety performance of and organizations. Specific actions related to safety participation are, giving suggestions and feedback to enhance safety, encouraging others to learn, act and perform safety, actively learning and participating in safety training, highlighting the possible safety-related issues to organizations, and stewardship behavior (62). Safety participation is an extra-role behavior that is voluntary (63).

We adopted the definition and dimension of safety behavior developed by Griffin and Neal (61). Prior literature in high-reliability organizations or high-risk industries affirmed that safety compliance and safety participation can be associated with occupational accidents (60, 63, 64). For example, the relation of safety compliance with safety procedure and near-miss was negatively related (65). Another researcher identified that prosocial safety behavior and proactive social behavior can also reduce the number of accidents (66). There is no doubt about the risk associated with the construction industry, safety compliance and safety compliance can perform a pivotal role in accidents and injury reduction (67, 68). All in all, research on these two dimensions of safety behavior in the construction industry may contribute meaningfully to the enhancement of occupation safety. Through this research, PsyCap as an antecedent to safety behavior will be assessed for the construction industry of Malaysia.

### Psychological Capital and Safety Behavior

According to the literature, employees' performance at work, satisfaction, and organizational citizenship behavior can be predicted and positively influenced by PsyCap (23, 39, 69). For instance, Avey et al. (23) highlighted that job satisfaction and positive outcomes can be achieved through PsyCap. According to Luthans et al. (17), the effect of sub-dimensions of PsyCap may vary on performance outcomes, satisfaction with the job, happiness at work, and organizational commitment e.g., hope, as it may have a stronger impact than the rest of other dimensions. As it is widely accepted that PsyCap positively influences job performance, but its effect on safety performance may result in showing different patterns, because of the contextual difference in the task and their applicability (60). There are different views about the direct and indirect effects of PsyCap on safety performance. Some state that PsyCap has a direct effect on performance (18), whereas others suggest that PsyCap has an indirect on safety through the mediation of motivation (60). Contrary to aforesaid, some scholars believe that PsyCap may have both direct and mediated effects on performance (70).

It has been proven through research that the greater exhibition of PsyCap can result in stronger individuals who can handle difficulties (71). To elaborate on theoretical assumptions associated with PsyCap, we will be opting for Social Cognitive Theory, coined by Bandura's (72) propositions. Social Cognitive Theory states that behavior is not only modified or influenced by the environment but can be affected through an individual's psychological perception, which partially relies on an individual's characteristics (73). PsyCap is a unique resource that is based on an individual's positive psychological state. As a result, this unique ability of the individuals helps them generate positive behavior and attain acceptable performance. Being a prominent sign of an individual's psychological situation, PsyCap can be defined as one's “self-evaluation.” Keeping aforesaid in view, it is defensible to say safety behavior, one of the components of work-related behavior will also be influenced by employees' PsyCap, and thus the following hypotheses are formed to be tested:

H1 Hope is positively associated with safety compliance and (H2), safety participation.
H3 Self-Efficacy is positively associated with safety compliance and (H4), safety participation.
H5 Resilience is positively associated with safety compliance (H6), safety participation.
H7 Optimism is positively associated with safety compliance (H8), safety participation.

### Psychological Capital and Work Engagement

PsyCap is a multidimensional construct that can be linked with a variety of variables. Hope is one of the dimensions that is widely known to be goal pursuance which resembles the engagement dimension called vigor (74). According to literature, hope is not something that acts as a contributor toward work engagement, but it becomes essential to have some, where its absence may lead to confused employees. Posited by Maslach et al. (75), burnouts are the main outcome of employee low hope, where individuals with no hope will face a deficient amount of willpower to embrace a new challenge, resulting from difficulty in finding a way out in difficult situations. The second dimension is self-efficacy, where efficacy, can be described as an employees' conviction or belief in one's self about the capability to deploy their motivation and psychological resources to successfully execute tasks at hand (76).

Prior studies have examined and demonstrated the direct as well as the indirect influence of self-efficacy on job engagement, indicating the path that individuals' involvement takes, hence PsyCap is one of the determinants of work engagement (77, 78). Optimism, which reflects individuals' capacity to see the bright side of the current and future events and connect them to performance results. According to the literature, cynical people are less optimistic, but optimism can assist reduce the effect of cynicism and increase devotion, as well as reduce the negative impact of various stresses (51, 79). It is the presence of optimistic ideas about a positive conclusion in one's mind that allows him/her to be more psychologically open, allowing the individual to absorb its surroundings and, as a result, lead to a higher degree of engagement (80). In general, optimism relates more to engagement components like dedication and absorption (74).

Luthans (42), described resilience as the individual's capacity to react concerning abrupt or significant circumstances. Whereas, the job demand resource model attributed resilience with persistence. Psychological resources act as a repository that provides resources like resilience for motivation and works engagement, which depicts an individual's vigor or robustness (74). According to the literature, resilience can work as a backup or extra source which can mitigate the excessive negative impact of job demand and burnout. Resilience can be known as one's state which can not only influence the present moment but also help to neglect past stress. Relatedness of resilience with work engagement is proportional, where one's resilience is increased at one side, it would help tackle job demand, stress, and overall control. With this in mind, it is reasonable to assert that resilience is linked to the characteristics of job engagement. Based on the preceding reasoning, it is reasonable to conclude that persons who utilize their PsyCap will achieve good performance, resulting in better work engagement (74), thus the following hypotheses are formed to test the PsyCap effect on work engagement.

H09 Hope is positively associated with Work Engagement.
H10 Self-Efficacy is positively associated with Work Engagement.
H11 Resilience is positively associated with Work Engagement.
H12 Optimism is positively associated with Work Engagement.

### Work Engagement and Safety Behavior

There is some empirical and qualitative evidence suggesting the relationship between engagement and work performance in general. For instance, engagement was associated with context and task performance (81). According to prior research, work engagement has proved to be positively influencing workplace outcomes e.g., organizational commitment, satisfaction in life, and organizational citizenship behavior (82–85). It has also been proved through research that employees who are more engaged, tend to see their job more positively and try to be productive, and are more interested in acquiring new knowledge (74). There is continuous arousal for engaged employees which keeps the spark alive for goal setting and attainment. Regarding safety performance, employees who show more engagement with work are more willing to exhibit safety behavior (86). Wachter and Yorio (87) highlighted that engaged employees perform role-specific activities and safety behavior as well. One of the important aspects highlighted by Sulea et al. (34) is that engagement at work results in the utmost dedication and pushes employees to go beyond their normal call of duty or routine work. This aspect of work engagement is quite similar to the safety participation role, which is voluntary and initiated by employees of their own free choice. Engaged employees are more eager to participate in safety because they have higher self-esteem and self-satisfaction. Evidence from previous literature also supports that work engagement is positively associated with safety outcomes (86, 88–90). Based on this discussion, we propose that work engagement will positively mediate the safety behavior of construction workers through the following hypotheses:

H13 Work Engagement is positively associated with safety compliance.
H14 Work Engagement is positively associated with safety participation.

### Mediating Role of Work Engagement

**Figure 1:**
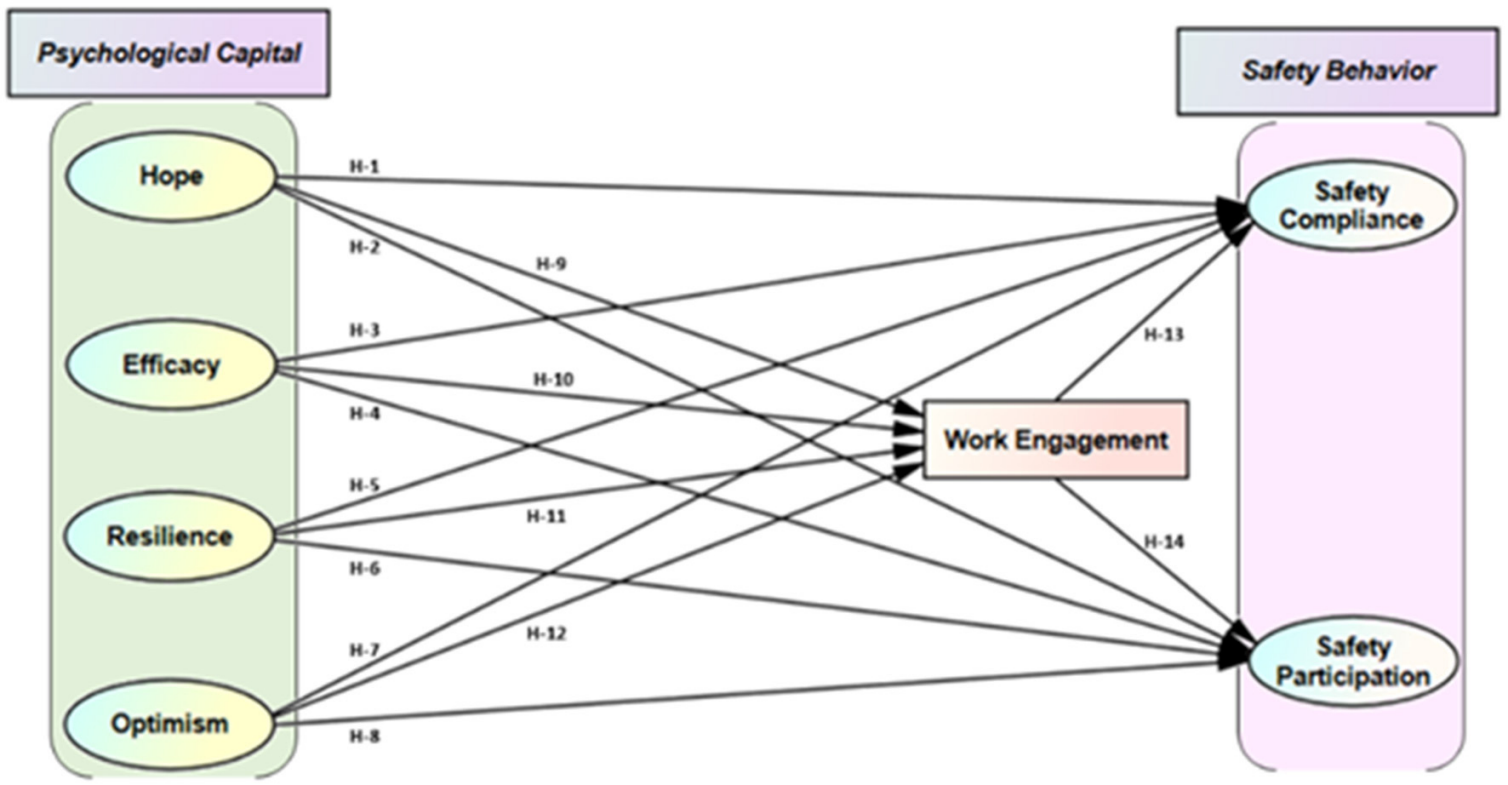
Hypothesized model.

There is much to explore about the fruitfulness of work engagement and its related outcomes. Where, the engagement itself is the outcome of resources (provided by the organization) (34), as well as resources held by employees in terms of their traits or characteristics. Prior literature identified PsyCap as a personal resource that has a significant impact on work engagement and it was supported that PsyCap contributes to increasing individual engagement at work (91). Leaving aside the personal resource component, it has been well-investigated and understood that if firms give adequate resources to employees, this, in turn, generates job engagement, which leads to organizational commitment and proactive behavior (34). Considering both ends of work engagement i.e., input (organizational and personal resources) output (organizational commitment, satisfaction in life and organizational citizenship behavior, positive emotions, vigor, dedication, and absorption), we hypothesize that work engagement will mediate the relation between all sub-dimensions of PsyCap and safety behaviors. Figure 1 depicts the graphical representation of the overall hypothesis of this study:

## Methods and Materials

### Research Design

The research design is the actual action plan that begins with the research questions and ends with the conclusion, as well as the debate and justification that follow, all of which operate simultaneously (92). Some of the study design includes quasi-experimental, longitudinal, and cross-sectional research designs; panel/cohort research designs; and comparative research designs (93). For this study, because all the data was collected at a certain time moment in time, thus this study utilized a cross-sectional research design. A study of the research phenomena and selected variables of our interest was possible using this method of data collection. Accordingly, basic features of an analysis unit (or unit of analysis), respondents (or individuals), sample strategy, data collection (or data gathering), and analysis will be discussed in later sections. Further, our study followed the quantitative paradigm that is in line with the cross-sectional research approach. The reason for opting for the positivist research paradigm is that this paradigm proposes the concepts that are established on facts and figures to better understand and appreciate current reality (94).

### Data Collection

The target population for this research was Malaysian construction workers who are at the operative level i.e., working at the proximity of the hazards or they are being the immediate recipient of the possible accident. We opted for non-probability convenience and show-ball sampling technique for our research as we do not have the exhaustive list of our entire population being studied (95, 96). Questionnaires were printed and hard copies were given to workers involved in construction activities e.g., roofers, masons, plumbers, tiles and bricks installers, ironworkers, electricians, pipefitters, and concrete finishers. Questionnaires were usually filled during work intervals, meal brakes and whenever the workers were available during their working hours. Respondents were assisted when they were giving their responses by assisting them to use their thoroughness and answer questions on their own. After completion, they returned the questionnaire to the research team on the ground. It took 15–20 min to fill the survey. Anonymity is the most important aspect while doing any survey and respondents were assured about the privacy and anonymity of their given responses. Respondents were also ensured that the research was conducted for academic purposes only.

### Data Analysis

For this study, data analysis was carried out in three different stages through IBM SPSS Version 21, IBM SPSS AMOS Version 21 software. Primarily, we analyzed descriptive and reliability statistics through SPSS 21. Thereafter, confirmatory factor analysis (CFA) was performed using AMOS 21 to test the validity of dimensions i.e., discriminant and convergent validity. For hypothesis testing, SPSS Amos 21 was used. Finally, a structural equation modeling (SEM) with 5,000 bootstrapping tests was conducted to assess the direct and indirect effect of PsyCap on safety behavior as well as the mediated effect of work engagement on safety behavior, an analysis technique commonly utilized (97–100). The measurement model, as well as the structural model, were measured and assessed through different goodness of fit (GOF) indexes e.g., *absolute fit indices* (how well the prior model fits or reproduces the data); RMSEA (root-mean-square error of approximation), GFI (goodness-of-fit index), and AGFI (adjusted goodness-of-fit index), *incremental fit indices* (deviation from the null model of representing factor); NFI (Normed fit index), TLI (Tacker-Lewis index), and CFI (Comparative fit index), and lastly the parsimonious fit indices (assessment of competing model); X^2^ (i.e., X^2^/degree of freedom) (101). Through aforesaid indices were utilized to assess the multifactor structure of PsyCap, safety performance, and unidimensional structure of work engagement.

### Questionnaire Design

There were four different sections for the questionnaire of this research. Those sections were respondents' demographic information, PsyCap, Work engagement, and safety behaviors. Initially, all of the questions/items were discussed with four field research experts from academia. The questionnaire was also shown and discussed with construction industry experts. The purpose of this was to assess whether questions are easily comprehendible and the overall structure is easygoing for the respondents to address face validity. No major changes were made in the instrument structure, where necessary, wording and text were reviewed and thoroughly studied following the conversational norms and habits of the construction site workers. Initially, four workers were met by the researcher along with an instrument to assess the content validity by discussing the questionnaire with them and observing if they were able to understand the written content.

The average time for questionnaire response was calculated through four respondents. To further enhance the reliability of the responses, overall four questions were reversed randomly, as it helped us in decreasing the socially desirable responding effect (102). For PsyCap, we used a 5-point Likert scale ranging from 01 “strongly disagree” to 05 “strongly agree.” For safety behavior, we also used the Likert scale, and respondents were asked to rate their safety performance through preference like 1 “never” to 5 “quite often.” For work engagement, a 7-point Likert scale ranging from a Likert scale, from 1 (strongly disagree) to a 7 (strongly agree) scale was used to measure employees' work engagement.

### Measures Used

The measure developed by Luthans et al. (39) for PsyCap was used for this study. Utilization of this questionnaire can be observed in various studies (15, 23, 43, 46, 78, 91) especially in the context of the construction industry. PsyCap has four distinct sub-dimension i.e., hope (perseverance to achieve the goal and align when necessary), self-efficacy (confidence in one's self and exerting efforts to succeed), resilience (tendency to bounce back after advertises), and optimism (looking at the bright side through positive attributes). The questionnaire was comprised of a total of twenty-four statements, whereas six statements were assigned to each sub-dimension. example of the statement is “I feel confident analyzing a long-term problem to find a solution,” “I usually manage difficulties one way or another at work,” “When things are uncertain for me at work, I usually expect the best,” “There are lots of ways around any problem.”

For work engagement, a scale developed by Schaufeli et al. (48) was used. This scale is a short version of work engagement, which is comprised of nine items. Three dimensions of work engagement are represented through this unidimensional scale e.g., vigor (greater energy level with enough mental resilience), dedication (inspiration and enthusiasm about one's work), and absorption (well-connected and engrossed with work throughout). For the work engagement scale, we used a 7-point Likert scale. Some of the question statements were, “At my job, I feel strong and vigorous,” “I am proud of the work that I do,” and “Time flies when I am working.”

Safety behavior was assessed using one of the renowned instruments developed by Neal and Griffin (64). This scale contains six items including three items for safety compliance (obligatory safety behaviors at the workplace, required formal compliance) and three statements for safety participation (extra-role behavior, not essential to perform, but self-generated behavior in the form of contextual performance). Statements for safety compliance are “I use all the necessary safety equipment to do my job,” “I use the correct safety procedures for carrying out my job, “and “I ensure the highest levels of safety when I carry out my job,” statements for safety participation are “I promote the safety program within the organization,” “I put in extra effort to improve the safety of the workplace” and “I voluntarily carry out tasks or activities that help to improve workplace safety.”

Scale for PsyCap and work engagement were adapted according to our research context i.e., construction industry, as the original scales items were generic and not-context specific to our research. We added the connecting statement before the original scale item as highlighted in previous literature as “item alteration” (103). An example of the adapted statements between PsyCap and work engagement is “I am able to positively engage in my work because; I feel confident in analyzing a long-term problem to find a solution” and “I am able to positively engage in my work because; I feel confident in representing my work area in meetings with management.” An example of the adapted statement between PsyCap dimension and safety performance is “I am able to exhibit safe behavior at the construction site because; there are lots of ways to solve any problem.” Lastly, the example statement between work engagement and safety performance is “I am able to generate positive safety behavior because; at my work, I feel bursting with energy” and “I am able to generate positive safety behavior because; I am enthusiastic about my job.”

## Results

### Demographic Results of the Research

Four hundred fifty questionnaires were filled through 25 ongoing construction projects in six provinces i.e., Kelantan, Johor Selangor, Perak, Penang, Negeri Sembilan of west Malaysia. Out of 450 questionnaires, 407 questionnaires were filled. After the assessment, 62 questionnaires were declared invalid because of inappropriate response marking, missing data, and obvious/odd patterns of responses. The final data was 345 valid questionnaires, which is 76.6% of the overall questionnaire disbursed. The details of the respondents are shown in Table 1.

**Table 1:** Demographic information of the respondents.

**Main category**	**Sub-category**	**Frequency**	**% of total responses**
Gender	Male	294	85
	Female	51	15
Respondent age	18–25	74	21
	26–35	124	36
	36–45	85	25
	45 & above	62	18
Work Experience	<5 year	81	23
	6–10 years	117	34
	11–15 years	86	25
	20 & above years	61	18
Education	Primary	37	11
	Lower secondary	63	18
	Upper secondary	82	24
	Post-secondary	97	28
	Diploma	66	19
States	Perak	64	19
	Johor	48	14
	Kelantan	65	19
	Negeri Sembilan	49	14
	Penang	58	17
	Selangor	61	18

### Reliability and Validity Analyses

To test the internal consistency of each construct of the questionnaire, a reliability test was performed. To achieve acceptable reliability in responses, the acceptable value of Cronbach's alpha is 0.70 (104, 105). For all of the seven constructs, Cronbach values ranged from 0.86 to 0.97 (Table 2), which shows that our results are robust and reliable.

**Table 2:** Convergent validity and reliability results.

**Constructs**	**Items**	**SFL**	**CR**	**AVE**	**α**
Hope	H1	0.814	0.92	0.657	0.92
	H2	0.780			
	H3	0.857			
	H4	0.809			
	H5	0.809			
	H6	0.793			
Efficacy	EF1	0.742	0.894	0.585	0.89
	EF2	0.747			
	EF3	0.786			
	EF4	0.782			
	EF5	0.786			
	EF6	0.745			
Resilience	RE1	0.733	0.883	0.557	0.88
	RE2	0.797			
	RE3	0.774			
	RE4	0.716			
	RE5	0.713			
	RE6	0.744			
Optimism	OP1	0.755	0.867	0.521	0.87
	OP2	0.726			
	OP3	0.709			
	OP4	0.695			
	OP5	0.696			
	OP6	0.748			
Work engagement	WE1	0.716	0.907	0.521	0.91
	WE2	0.715			
	WE3	0.723			
	WE4	0.746			
	WE5	0.706			
	WE6	0.719			
	WE7	0.712			
	WE8	0.729			
	WE9	0.730			
Safety compliance	SC1	0.973	0.97	0.92	0.97
	SC2	0.957			
	SC3	0.949			
Safety participation	SP1	0.927	0.94	0.83	0.94
	SP2	0.902			
	SP3	0.907			

*SFL, standardized factor loadings; CR, construct reliability; AVE, average variance extracted; α, Cronbach value*.

There are common indicators like construct reliability (CR), standardized factor loadings (SFL), and average variance extracted (AVE) to measure and assess the discriminate validity of the construct being studied. The critical value for aforesaid parameters are, SFL > 0.6, CR > 0.7, and AVE > 0.5 (98, 106). Results of the convergent validity indicators are also shown in Table 2, which is reflecting the appropriate power of each item concerning its variables, whereas all variables met threshold criteria, thus demonstrating an acceptable convergent validity. For discriminant validity, the square root of the average variance extracted (AVE) value was compared with the correlation coefficient of other variables (107, 108). Further, if the outcome value is greater than its correlation coefficient then the discriminant validity is achieved (reflected in Table 2). All of the constructs met this criterion and are depicted in Table 3.

**Table 3:** Discriminant validity results.

**Constructs**	**AVE**	**Hope**	**Efficacy**	**Resilience**	**Optimism**	**Safety compliance**	**Safety participation**	**Work engagement**
Hope	0.66	0.811						
Efficacy	0.59	0.133	0.765					
Resilience	0.56	0.215	0.284	0.747				
Optimism	0.52	0.255	0.075	0.047	0.722			
Safety Compliance	0.92	0.341	0.056	−0.141	0.21	0.96		
Safety Participation	0.83	0.395	0.468	0.471	0.633	0.261	0.912	
Work Engagement	0.52	−0.41	0.136	−0.104	0.028	0.204	0.114	0.722

### Measurement Model

To assess the validity of the measurement model for PsyCap and work engagement constructs, confirmatory factor analysis was performed. Our result indicated a good fit for measurement mode (109). A few of the indices like CMIN (chi-square X^2^/degree of freedom), chi-square X^2^, comparative fit index (CFI), root-mean-square error of approximate (RMSEA), normed fit index (NFI), goodness-of-fit index (GFI), adjusted goodness-of-fit index (AGFI), Tacker-Lewis index (TLI) (110, 111) were used to assess the measurement model fit for both one factor model of PsyCap, four factor model of PsyCap and one factor model of work engagement. The quality of the one factor model of two measurement models (PsyCap and work engagement) was confirmed, whereas, all of the values for each index were well under the criteria. Results for four fist order factors (primary factors) for PsyCap were; (Hope; *P* = 0.012, RMSEA = 0.062, GFI = 0.981, NFI = 0.985, AGFI = 0.955, CFI = 0.991, TLI = 0.985, and CMIN = 2.38), (Self*-*efficacy; *P* = 0.935, RMSEA = 0.00, GFI = 0.997, NFI = 0.997, AGFI = 0.992, CFI = 1, TLI = 1.00, and CMIN = 0.402), (Resilience; *P* = 0.212, RMSEA = 0.032, GFI = 0.990, NFI = 0.989, AGFI = 0.974, CFI = 0.997, TLI = 0.995, and CMIN = 1.352), and (Optimism; *P* = 0.513, RMSEA = 0.00, GFI = 0.992, NFI = 0.99, AGFI = 0.982, CFI = 1, TLI = 1.002, and CMIN = 0.912), which showed the best fit model fit for all the dimension of PsyCap.

Table 4 represents the overall one-factor model fit statistics against the confirmatory factor analysis (CFA), which shows that our results are acceptable and a good fit was achieved for the measurement model of PsyCap and work engagement. To summarize the scales' reliability, convergent validity, and discriminant validity, our study findings support that the internal factor structure of all scales being tested is well-validated and reliable by meeting the convergent validity as well as discriminant criterion with associated variables (112). These findings add further value to the efficacy and predictability of the instruments being utilized in this study.

**Table 4:** Fit indexes for the measurement models.

**Categories of statistics**	**Statistics**	**Fitness criteria**	**Psychological capital**		**Work Engagement**	
			**Value**	**Decision**	**Value**	**Decision**
Absolute fit indices	RMSEA	<0.08	0.003	Accept	0.004	Accept
	GFI	>0.90	0.944	Accept	0.983	Accept
Incremental fix index	AGFI	>0.90	0.932	Accept	0.972	Accept
	NFI	>0.90	0.946	Accept	0.983	Accept
	TLI	>0.90	1	Accept	1	Accept
	CFI	>0.90	1	Accept	1	Accept
Parsimonious fit indices	χ^2^/DOF	<2.00	1.003	Accept	0.959	Accept

*RMSEA, root-mean-square error of approximation; GFI, goodness-of-fit index; AGFI, adjusted goodness-of-fit index; NFI, Normed fit index; TLI, Tacker-Lewis index; CFI, Comparative fit index; PNFI, Parsimony normed-fit index; X^2^ (i.e., X^2^/degree of freedom)*.

### Structural Model

We formulated the hypothesized model (Figure 1) and with the SEM technique. To assess the Goodness-of-fit, we tested if the responses fitted the measurement as well as a structural model. Concerning the criteria for structural model fit (107), primarily model fit was seen to see if there are any abnormal variables, where all variance was significant with a value >zero, standard errors were well under the limit and all standardized factor loading were significant with the value ranging from 0.69 to 0.97. Our results exhibit strong empirical evidence for the good primary fit of the data. Again for the second phase of the overall model fit for all variable, we used indexes such as; absolute, incremental, and parsimonious (110), and values against criterion indexes for the overall model is in Table 5. All the indexes met the criteria, demonstrating an acceptable overall model fit.

**Table 5:** Fit indexes for the structural model.

**Categories of statistics**	**Statistics**	**Fitness** **criteria**	**Overall model**	
			**Value**	**Decision**
Absolute fit indices	χ^2^	–	857.582	Accept
	RMSEA	<0.08	0.028	Accept
	GFI	>0.90	0.887	Accept
	AGFI	>0.80	0.87	Accept
	NFI	>0.90	0.909	Accept
	TLI	>0.90	0.978	Accept
Incremental fix index	CFI	>0.90	0.979	Accept
Parsimonious fit indices	χ^2^/DOF	<2.00	1.263	Accept

*RMSEA, root-mean-square error of approximation; GFI, goodness-of-fit index; AGFI, adjusted goodness-of-fit index; NFI, Normed fit index; TLI, Tacker-Lewis index; CFI, Comparative fit index; PNFI, Parsimony normed-fit index; X^2^ (i.e., X^2^ /degree of freedom)*.

### Hypothesis Testing

For hypothesis testing, we use a 95% confidence interval, whereas if the *P*-value of any variable is under 0.05 with the positive estimate, it would lead to acceptance. At first, the direct effect of PsyCap's sub-dimensions on safety behavior (safety compliance and safety participation) was tested. The outcome of the hypothesis testing depicted that both hypotheses (H1) (β = 0.524, *p* < 0.001), and (H2) (β = 0.231, *p* < 0.001) of Hope toward safety compliance and safety performance were accepted. For self-efficacy, (H3) (β = −0.01, *p* > 0.05) was rejected based on its non-significant and negative association with safety compliance, but for self-efficacy (H4) (β = −0.277, *p* < 0.001) toward safety participation was accepted. Out of two hypotheses for resilience, (H5) (β = −0.205, *p* < 0.001) was rejected based on negative association, whereas (H6) (β = 0.335, *p* < 0.001) of resilience with safety participation was accepted. Pertinent to the last sub-dimension i.e., optimism, both hypotheses (H7) (β = 0.089, *p* < 0.001) and (H8) (β = 0.533, *p* < 0.001) were accepted for safety compliance and safety participation.

Secondly, all sub-dimensions of PsyCap were tested with work engagement. Results indicated that Hope (H9) (β = −0.45, *p* < 0.001) had a significant but negative association with work engagement, whereas self-efficacy (H10) (β = 0.207, *p* < 0.001) had a significant and positive association with work engagement. Further, resilience (H11) (β = 0.207, *p* > 0.05) was found to have an insignificant effect on work engagement. Lastly, optimism (H12) (β = 0.128, *p* < 0.001) was found to have a significant and positive association with work engagement.

**Figure 2:**
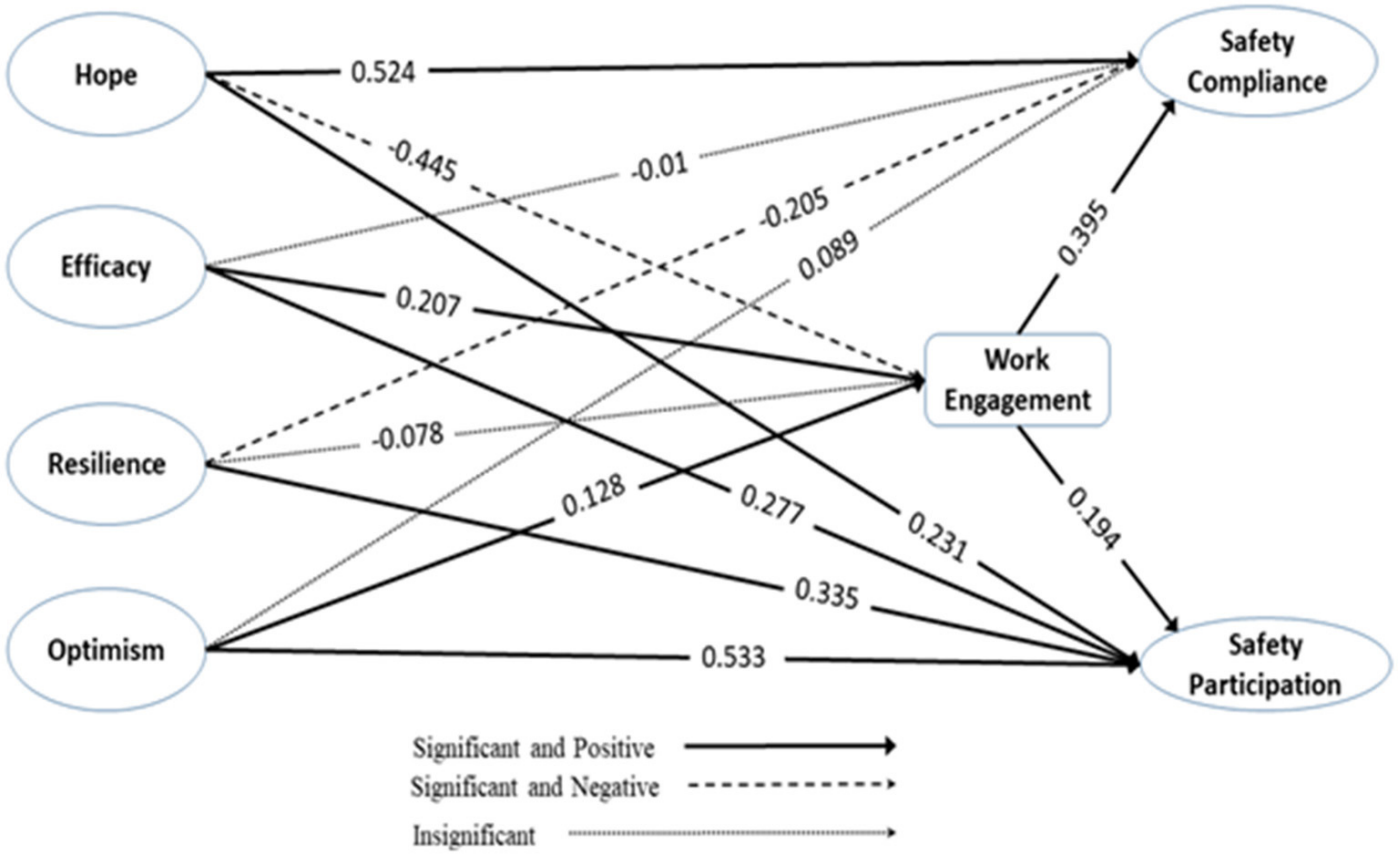
The final structural model and impact paths.

Finally, the direct effect of work engagement on safety behavior indicators i.e., safety compliance and safety participation to observe how work engagement impacts safety outcomes. As expected, both (H13) (β = 0.395, *p* < 0.001) and (H14) (β = 0.194, *p* < 0.001) were accepted as they showed a significant and positive association between work engagement and safety performance objective indicators (see Figure 2, Table 6). It was also found that PsyCap dimensions self-efficacy and optimism explained about 21.9% of the total variance in workplace engagement. Further, it was also found that 40% of the variance in safety compliance was explained by hope, optimism, and work engagement, while 73.8% of the variance in safety participation was explained by hope, efficacy, resilience, optimism, and work engagement. The amount of variance explained by the predictors of safety compliance i.e., hope, optimism, and work engagement in this study shows the importance of these variables to enhance the safety compliance behavior of the employees. Also, the amount of variance explained for safety participation through the predictor variables showed a strong association, indicating potential promising association.

**Table 6:** Path coefficients of the final model.

**Path**	**Estimate**	**S.E.**	**C.R.**	***P***	**Result**
Hope (H 01)	—>	Safety compliance	0.524	0.08	8.321	***	Accept
Hope (H 02)	—>	Safety participation	0.231	0.05	5.236	***	Accept
Efficacy (H 03)	—>	Safety compliance	−0.01	0.08	−0.184	0.85	Reject
Efficacy (H 04)	—>	Safety participation	0.277	0.06	6.6	***	Accept
Resilience (H 05)	—>	Safety compliance	−0.205	0.09	−3.735	***	Reject
Resilience (H 06)	—>	Safety participation	0.335	0.07	7.787	***	Accept
Optimism (H 07)	—>	Safety compliance	0.089	0.08	1.673	0.09	Accept
Optimism (H 08)	—>	Safety participation	0.533	0.07	11.2	***	Accept
Hope (H 09)	—>	Work engagement	−0.445	0.08	−6.935	***	Reject
Efficacy (H 10)	—>	Work engagement	0.207	0.09	3.486	***	Accept
Resilience (H 11)	—>	Work engagement	−0.078	0.1	−1.33	0.18	Reject
Optimism (H 12)	—>	Work engagement	0.128	0.09	2.196	0.03	Accept
Work engagement (H 13)	—>	Safety compliance	0.395	0.06	6.433	***	Accept
Work engagement (H 14)	—>	Safety participation	0.194	0.04	4.511	***	Accept

*** *p < 0.001; Estimate, standardized regression coefficients; S.E., standardized error; C.R., critical ratio*.

To test the mediating role of work engagement between PsyCap dimensions and safety behavior (safety compliance and safety participation), the bootstrapping technique was used and, the results are depicted in Table 7. Mediation results highlighted that work engagement played a partially mediating role between hope and safety compliance (β = −0.176, *p* < 0.001), and a fully mediating role between efficacy (β = 0.082, *p* < 0.001), and safety compliance. Further, the evidence of the partially mediating role of work engagement was also found between hope (β = −0.087, *p* < 0.001), efficacy (β = 0.04, *p* < 0.001), optimism (β = 0.05, *p* < 0.001), and safety participation. Nonetheless, there was an insignificant mediating effect observed between resilience (β = −0.015, *p* > 0.05) and safety participation, and resilience (β = −0.031, *p* > 0.05) and safety compliance.

**Table 7:** Standard direct and indirect effects for the mediation model.

**Mediation effect**	**Direct effect *X → Y***	**Indirect effect**	**Result**
Hope → Work engagement → Safety compliance	0.524**	−0.176**	Partial mediation
Hope → Work engagement → Safety participation	0.231**	−0.087**	Partial mediation
Efficacy → Work engagement → Safety compliance	−0.01(ns)	0.082*	Full mediation
Efficacy → Work engagement → Safety participation	0.277 **	0.04*	Partial mediation
Resilience → Work engagement → Safety compliance	−0.205**	−0.031 (ns)	No mediation
Resilience → Work engagement → Safety participation	0.335**	−0.015 (ns)	No mediation
Optimism → Work engagement → Safety compliance	0.089 (ns)	0.05 (ns)	No mediation
Optimism → Work engagement → Safety participation	0.533**	0.05*	Partial mediation

*ns, P > 0.05 (not significant)*;

* *P < 0.05 (Significant)*;

** *P < 0.01 (significant)*.

## Discussion

Our study was aimed to assess the impact of PsyCap on the safety behavior of construction industry workers while taking work engagement as a mediating variable. The empirical evidence was obtained between the discriminant facets of PsyCap i.e., hope, self-efficacy, resilience, and optimism against safety behavioral outcomes. Our results suggest that hope, optimism, and work engagement have a significant and positive impact on both safety compliance and safety participation. Further, efficacy and resilience were also found to have a significant and positive impact on safety participation. It was interesting and unexpected to find that resilience had a significant, but negative relation with safety compliance, hence showing that a more thorough and precise viewpoint to be given toward PsyCap adaption. Additionally, partial mediation of work engagement was found to reduce the negative effect of resilience on safety compliance. The direct effect of Psycap dimensions on workers' safety behavior and work engagement has been discussed separately in the following sections:

### Psychological Capital Direct Effect on Workers' Safety Behavior

#### Association Between Hope and Safety Behavior

Established by Luthans et al. (39), that hope is related to the positive outcomes at the workplace for employees, and in our case, our findings were in harmony with that. Hope was found to have a significant and positive effect on both indicators of safety behavior which is in contrast with some literature (46). Our findings are also in consensus with some of the prior findings (15). Although our findings suggested that hope is positively associated with safety behavior, prior researches highlighted certain elements like excessive remote deployment, staying far away from home, having a non-permanent job, demanding and hazardous nature of the job (2, 36), can modify the hope of construction workers. Other characteristics elements like diverse and non-permanent team members, stringent project timelines, etc. can also modify construction employee behavior (36), possibly impacting the hope of the individual. Subsequently, construction firms need to work upon such elements to reduce their possible negative effect on employees' hope. Our findings suggest that high hope is associated with enhanced safety behavior, which contradicts the findings of He et al. (46). As described by Luthans et al. (39) hope works through two mechanisms; willpower to achieve goals and way power to choose pathways to attain goals. It is also worthwhile to mention here that hope does not always enhance safety behavior as it is based on individual willpower and excessive willpower to attain certain tasks and goals may undermine the safety behavior, thus a balance between willpower and task is necessary (113). In general, hope is one of the optimistic states of an individual, which is expected to motivate individuals for positive outcomes, thus our findings are consistent with this expectation.

#### Association Between Self-Efficacy and Safety Behavior

Bandura (114) posited that self-efficacy can affect one's feelings and motivation to engage in certain behavior. In our case, self-efficacy was found to be significantly impacting safety participation only, which is in harmony with prior literature (15, 46, 115), but self-efficacy was found to have an insignificant association with safety compliance which is unexpected and needs further validation. Keeping the intrinsic feelings of construction workers in mind, it is expected that self-efficacious workers tend to have more control over their working environment, thus resulting in better safety performance. In congruence with the propositions of the Theory of planned behavior (116), which stated that social and personal factors influence an individual's behavior and self-efficacy shows the potential to be applied as a predictor of an individual's safety behavior according to our findings. The utility of self-efficacy is not just limited to safety, but it also helps individuals to produce better outcomes at work in other contexts (117), thus continuous utilization of self-efficacy by construction workers may yield more stable safety results. Under the light of Bandura's assumption (72) that, it is the individual's judgment about his/her ability to manage the ongoing situation through the prepossessed skill, we can say that when a construction worker is faced with a safe situation, his/her role may conflict, thus leading to the non-compliance. This may have been caused by performance pressure (quantitative overload), e.g., a production supervisor is supposed to produce higher as well as to prioritize safety over productivity, which itself is a contradiction (118). Literature also states that highly efficacious individuals perceive demanding situations as more challenging, as they invest their energies and time against those situations, and in doing so they feel themselves less vulnerable to the outcomes (114). Consistent with aforesaid, one of the characteristics of construction workers' work behavior is having mastery in a certain task, leading to the possible excessive efficacious belief, because having prior experience in the same task may have undermined their safety compliance. Additionally, in our case, the employee may have found themselves efficacious to show participatory behavior, but their job/role demand might disrupt their compliance behavior, which yet requires more validation in future to testify this research outcome.

#### Association Between Resilience and Safety Behavior

Prior literature suggests that resilience is expected to have a positive effect on safety behavior (15, 39, 46), In our case, resilience was found to have a significant and positive effect only on safety participation, but a significant and negative effect on safety compliance, which is unexpected. Malaysian workers usually come from rural areas and they usually inherit the profession of agriculture, where agriculture accounts for a 7.1% contribution to national GDP, making it one of the significant sectors (119). One can expect that workers have to learn new skills, adapt to ever-changing living conditions, work pressure, and other personal factors (36), which might affect their compliance behavior. Posited by Olumide and Owoaje (120) in their study conducted on “Rural-urban disparity in knowledge and compliance with traffic sign” another form of “safety compliance,” that individuals from rural areas tend to ignore safety compliance which can be the outcome of less knowledge about regulation and standard operating procedures. In connection with this, construction workers need to be more informed by their employers through training, discussion, and demonstrations which could enhance their knowledge and exposure toward safety compliance.

Another positive aspect of the positive relationship between resilience and safety participation is that workers who are coming from remote areas and backgrounds tend to be more resilient as they may have learned a lot from their prior experience and through their transitions to different locations, which have added value to their knowledge, thus encouraging them to show a positive attitude toward safety participation. It is obvious to see that individuals living on their own must be resilient as they have to face unpredicted situations and they expect to have less support from their surroundings, as these are also the occupational characteristics that the construction sector holds. This is high time for businesses to wholeheartedly focus on acts that can enhance the resilience of their employees, as this quality of individuals does not only help themselves, but it will also help organizations to recover from the worst scenarios. For instance, if an organization is facing financial hardship, fierce competition, and another constraint, it will ultimately put pressure on its employees in terms of overload, financial cut-downs, and so forth. In this scenario, a resilient workforce would be of prodigious help for organizations to sustain those hard times. Talking about the prime characteristics of the construction sector i.e., uncertainty and unannounced crisis often, not just physically but financially as well (121), this sector itself needs to be resilient as this sector is essential for the overall society. The organizational ability to be resilient toward unforeseen situations can be uplifted with the workforce that is more resilient and able to manage uncertainties. It is evident to observe positive outcomes of resilience especially for occupational safety, thus it allows researchers to explore this variable in other organizational settings.

#### Association Between Optimism and Safety Behavior

Optimism was significantly and positively associated with both safety compliance and safety participation, which is consistent with the findings of some studies (15, 122) and in contrast to the findings of certain literature (46). It is a worthy indication that optimism is positively influencing safety participation, and it shows that the construction workforce is optimistic about the outcome they expect from their actions. This exhibition of optimism can negate the negative effect of a pessimistic approach not only toward safety but also toward other work domains, as pessimism is contagious, negatively affecting the attitude and behavior of not only certain individuals but other employees also (123). There is also a tendency that workers might have an inflated or unrealistic optimism (124, 125), which possibly affects safety outcomes, in other words, too much optimism about the behavior related to safety can also cause accidents and derail the safety programs. Evidence from the Australian construction industry suggests that optimism bias was not related to deteriorated occupational performance and it could be due to the social desirability bias of their respondents (124). Certain factors of the construction sector i.e., safety climate, work condition, individual's safety perception, cognitive bias, and risk perception can influence his/her optimism (54). Posited by Perrow (126), accidents are normal and are formed through systematic failures, and keeping this assumption in mind, one should not be too much optimistic about the prevailing safety instructions, scenarios, and actions at hand, but try to look far beyond. For the construction industry, the optimism construct needs to be utilized with more consideration and concentration.

In summary, the association of PsyCap was evaluated with safety behavior, where most of the sub-dimensions of PsyCap were positively and significantly associated with safety behavior, except efficacy and resilience with (safety compliance). The idea of PsyCap in connection with safety behavior may be communal, yet it still required more empirical evidence to achieve generalizability. While applying PsyCap in work and safety-related mechanisms, one should keep in mind the possible unexpected role of PsyCap.

### Mediating Effects of Work Engagement

Work engagement partially mediated that relationship between; hope and safety compliance (Hope → Work Engagement → Safety Compliance), hope and safety participation (Hope → Work Engagement → Safety Participation), self-efficacy and safety participation (self-efficacy → Work Engagement → Safety Participation) as well as optimism and safety participation (Optimism → Work Engagement → Safety Participation). Work engagement fully mediated the relationship between self-efficacy and safety compliance (Efficacy → Work Engagement → Safety Compliance). There was no mediating effect of work engagement between; resilience and safety compliance, resilience safety participation as well as optimism and safety compliance. Although work engagement mediated the relationship between hope and safety behavior, it tends to have a negative indirect effect on safety behavior which is unexpected and against the literature (91, 127). A possible justification for this situation could be the excessive engagement shown by employees, as engagement at work goes beyond the normal call of duty (34). The excessive drive to be more productive via work engagement may force an employee to ignore personal and occupational safety to pursue their goals. Although prior literature supported the assumption that engaged employees are tended to be safe employees, this assumption needs more empirical evidence, as in our case the findings were contradictory. Organizations must design interventions that are directly related hope with the safety phenomenon, rather than going for the indirect interventions.

An efficacious attitude of individuals can result in engagement (128), where there was no mediation effect between efficacy and safety compliance that is in contrast with prior findings (127), and there found partial mediation between efficacy and safety participation. One of the possible reason could be the nature of the outcome as safety behavior are the subjective outcome, and not the objective, individuals tend to involve in such behavior of their own will and choice. As construction workers face tough and hazardous working conditions, thus they require strong self-confidence and motivation to perform. If workers tend to lack self-efficacy, they may not be more involved, thus leading to unsafe behavior. Future researchers should assess this relation in different contexts and organizations should design interventions that can foster the self-efficacy of an individual.

One of the interesting findings of this research was the full mediation effect between self-efficacy and safety compliance. This implies that safety compliance can only be influenced by the inclusion of engagement as a mediator between efficacy and safety compliance. The appropriate level of self-efficacy can influence individuals to observe safety compliance which ultimately will lead to enhanced safety performance. It was also noteworthy to find that there was no mediation of work engagement between resilience and safety behavior which is out of the ordinary as work engagement is expected to produce positive work outcomes (128). Out findings are contradicting with prior literature (129, 130), which requires further assessment. There may be cultural and contextual differences that might have affected the mechanism of efficacy and work engagement or the industrial characteristics. Lastly, work engagement partially mediated the relationship between optimism and safety participation. This implies that optimistic individuals tend to be more productive and engaged, as they foresee and expect positive outcomes. Intervention addressing the relationship between optimism and work engagement might further strengthen the safety outcomes for organizations. The further mediating role of work engagement between optimism and safety compliance was insignificant, although the linear effect of optimism on safety compliance was positive and significant. Overall our research findings are in congruence with few of the past studies that have tested PsyCap as the possible predictor of work engagement (26–28), and in our case, only two of the dimension of Psycap were found positively and significantly affecting workers' work engagement behavior. We expect to see different results in the future if the scale of Psycap can be utilized as unidimensional to predict the work engagement behavior of the construction industry workers.

### Theoretical Implications

Our study contributed to safety literature by assessing the impact of PsyCap on employees' work engagement and their safety behavior (safety compliance and safety participation). First, our study reported the sound psychometric properties of all the measurement scales that we utilized for this study. By doing so, we have obtained further validity of these scales in the construction industry context. The concept of PsyCap (17) has not been introduced to many industrial sectors, consequently, the validation of this scale, as well as its predictors and outcomes, are not abundant in the literature. The role of PsyCap in the purview of occupational safety especially in the construction industry is very limitedly studied except for a few studies (30, 53). Our study went one step further by not just assessing the direct effect of PsyCap dimensions on safety behavior, but also proposing the four dimensions of PsyCap as a possible predictor of work engagement. Referring to the role of PsyCap for safety performance, it is expected to be a key construct in the coming years. Work engagement refers to the individual's vigor, dedication, and absorption while performing tasks, and in our study work engagement exhibited a positive association with safety compliance and safety participation. One of the possible reasons for this could be the assumption that workers who are highly engaged in their work are more aware of their surroundings and are more likely to engage in safe behavior (30). Our findings also exhibited that work engagement possibly mitigates the negative effect of false employees' hope, which is of great importance to understanding individuals' hope. Further work engagement also depicted that it can further enhance the direct effect of self-efficacy for both safety compliance and safety participation as well as for optimism and safety participation.

Such findings of this study elaborated the notion of work engagement via empirical testing and exhibited its positive impact on both safety compliance and safety participation. Further, prior literature has identified many predictors of safety performance (53, 131, 132), whereas the combined empirical evidence of both PsyCap and work engagement on safety behavior was yet to be tested. In past, much of the research has been devoted to identifying and testing variables that are not intrapsychic rather social, hierarchical, and organizational, where our study is of great value, as it demonstrated the positive association between PsyCap certain dimensions, work engagement, and safety performance. Lastly, we tested the theoretical assumption of social cognitive theory, stating that an individual's behavior is not solely influenced by their environment, but one's psychological perception can also play its part as well as one's characteristics. This theoretical assumption was supported through this study as sub-dimension of PsyCap as well as individual's work engagement showed association in between. At last, our findings can enhance prevailing knowledge of the relationship between safety behavior and its antecedents, adding further breadth to the understanding.

### Practical Implications

Our findings suggested that PsyCap should be treated with much care and attention in an organization. Although optimism predicted positive outcomes for both safety behaviors, organizations must keep in mind that the bias in terms of the over-optimistic behavior may prevail, which can influence employees' indulgence in safety behavior. Organizations should strategize their systems and policies keeping this bias in mind. It must be made clear through an organizational strategy that organizational health and safety standards are universally applicable regardless of experience, gender, role, and hierarchical position. Another important implication of the PsyCap sub-dimension is a false hope that can probably ruin the safe behavior by ignoring possible adversities, thus deteriorating organizational safety performance. These aspects make PsyCap advantageous as well as sensitive for organizations who would like to opt for it. Although it is not possible to measure the exact amount of hope and optimism of an employee for a specific outcome, a balance for this would be expected to be beneficial for both workers and organizations. It would also be beneficial for managers in terms of their safety-related objectives if they can opt for PsyCap interventions depending on the employees' PsyCap level. Talking in terms of effect size, hope and optimism both had a greater influence on safety compliance and safety participation of construction workers, therefore relevant interventions for enhancing safety can be designed to alleviate workers' PsyCap. One of the basic interventions is introducing employee training and activities that are tailored to boot psychological health.

Considering the positive effect of work engagement on both safety behaviors, managers should take certain measures to enhance work engagement. For instance, vigor can be increased through exercise as well as focusing on the life of an individual (133). To increase dedication amongst employees, the first step is to know them as the needs and expectations of every individual are different. There is a plethora of literature that can highlight certain ways to improve employee dedication and managers of the construction sector can choose them according to their teams and other factors. Lastly, to enhance absorption amongst employees to enhance safety performance, managers should make their activities interesting and easy so that they become engrossed while performing. Reference to the personal characteristics of study respondents, education of the construction worker is pivotal for one's self-confidence and efficacy, thus managers should give construction work awareness about their roles and responsibilities clear. This may help in boosting their confidence and enhancing their efficacy. Finally, the resilience of the construction workers can be enhanced through portraying specific scenarios, discussing worst-case scenarios of past and training, where they may be introduced to the certain scenario to see their reaction and learn out of it.

Since the data was gathered from local firms, hence the global generalization of the findings is not recommended, however, a few points are mentioned here as organizations, in general, have some common characteristics. Nonetheless, considering the generalizability of the findings, we used a sample that was drawn from different construction firms with a diversified set of respondents having different experiences, positions, and backgrounds. There is a bundle of variables that are generalizable across organizational-level, but specifically talking about PsyCap, it contains immense opportunities for organizations. Under the context of positive organizational behavior, efforts to introduce PsyCap may trigger the development of self-development, self-regulation, and self-awareness amongst employees (39). To generalize the study findings of hope and optimism results regarding safety compliance and participation behavior, organizations should work upon setting up clear goals and pathways for their workforce, as it may help employees to have genuine hope, and optimism and may protect them from having false hope or being over-optimistic. Since the designing of career goals and removing obstacles while performing jobs is universal to all organizations as one of their function, it can be implied that our findings may help design strategies related to hope and optimism for employees in other industrial settings. To generalize the outcome of efficacy and resilience regarding participation behavior, in general, organizations do have a mechanism through which they try to persuade and arouse their workforce toward success, as well as appreciate those who have achieved the desired outcomes. some high-reliability organizations (HROs) also work upon establishing a mechanism that mitigates or avoid possible risks, and also a design mechanism that may negatively affect their process (100). Keeping this in view our findings can be generalized in other organizations to enhance participatory behavior since one's self-belief/confidence and being resilient would not help to perform an ordinary task, but it would also help him/her to go beyond the normal call of duty.

### Study Limitations

One of the prime limitations would be the utilization of cross-sectional data, which is widely used in the research world, whereas the longitudinal approach might yield more robust and reliable results in future studies. Secondly, we used self-reported measures for all of the study variables, and that might cause social desirability bias for the respondents, as they may have overrated their PsyCap, work engagement, and safety performance behavior. Further, his study is limited to the Malaysian cultural context, which requires further validation in different contexts. Further, our respondents were mainly workers, who are usually not highly qualified in terms of academic education, thus limiting the view to some extent. Therefore, research on a much wider level with diversified respondents would reveal more insights. As we found mediation evidence impacting PsyCap relation with safety behavior, thus more exploration in terms of individual evaluation of PsyCap, groups, and team may also yield different results. As this research is based on the construction industry workers, where employment for is mainly temporary or project-based, thus it may not unveil the complete picture or understanding of their behavior, therefore assessment of this framework in other hazardous industries where workers are employed over a length of time may provide different results. Another limitation of this is research is the distribution of our respondents, as most of the workers in the construction industry are male, thus including industries that have justified/optimum population mix may also provide different results.

## Conclusions

This research helps enrich the current body of knowledge on employees' safety behavior (safety compliance and safety participation) in connection with PsyCap (hope, self-efficacy, resilience, and optimism) and work engagement (vigor, dedication, and absorption. This research contributed to the body of knowledge through; (a) identifying the influencing role of PsyCap on construction workers' safety behavior and its possible mechanisms, (b) differentiating the individual effect of each sub-dimension of PsyCap on safety behavior indicators, (c) the partially and fully mediating role of work engagement between PsyCap and safety behaviors. The finding of our research would help better-operationalizing interventions of PsyCap and work engagement to enhance safety performance and reduce occupational injuries. It was highlighted that one should be careful while opting for PsyCap's different dimensions to enhance safety.

Our results suggest that hope and work engagement have a positive and significant influence on safety behavior. Moreover, efficacy, resilience, and optimism have a positive and significant impact on safety participation. Nonetheless, efficacy was found to have an insignificant impact on safety compliance, whereas resilience was found to be negatively affecting safety compliance behavior. Both of these findings are contradictory to prior knowledge and need further quantitative evidence. Results also suggested that efficacy and optimism are positive and significant predictors of work engagement leading toward safety compliance and safety participation. Contrary to this, resilience had non-significant and negative relations, as well as hope, had a significant but negative association with work engagement. The indirect effect of resilience and optimism being insignificant provides an opportunity for future researchers to test this phenomenon.

PsyCap is a sensitive tool, which requires an in-depth amount of care and attention when applied, where elements like efficacy and resilience can negatively affect safety behavior. On the other hand elements like hope and optimism can positively influence work engagement and safety behavior. Our study also resulted in the theoretical advancement for Social Cognitive Theory (134), which states that personal factor also influences individual choices. In our case, hope, efficacy, resilience, and optimism being individualistic personal cognitive capabilities showed their influence toward the behavioral outcomes of construction workers, hence verifying the theoretical propositions of Social Cognitive Theory. PsyCap and work engagement need to be applied with care. The good news for management science researchers is that work engagement intervention may be anticipated using self-efficacy and optimism, both of which are individualistic but can be molded and impacted by organizational policies to increase work engagement. In contrast, hope may have a big yet negative influence on job engagement, which must be carefully considered when deciding how to improve work engagement. Organizations should carefully examine the existing degree of individual PsyCap and work engagement behavior, and then plan interventions appropriately. Although it is tough for managers to assess their workers' PsyCap status and allocate responsibilities and assignments. Consideration must be given to individuals' PsyCap, i.e., what kinds of attributes are necessary to accomplish a specific activity.

## Data Availability Statement

Data of the research are only available through the corresponding author upon reasonable request.

## Ethics Statement

Ethical review and approval was not required for the study on human participants in accordance with the local legislation and institutional requirements. The patients/participants provided their written informed consent to participate in this study.

## Author Contributions

Idea inception and execution were done by MS and GN, led by content analysis and writing. AI and YY further refined the concept, revised, and reviewed the overall manuscript writing. All authors read and approved the final manuscript.

## Funding

ACP of this research is funded by the YUTP project, Universiti Teknologi PETRONAS. We are also very thankful to the management of Universiti Teknologi PETRONAS for supporting us.

## Conflict of Interest

The authors declare that the research was conducted in the absence of any commercial or financial relationships that could be construed as a potential conflict of interest.

## Publisher's Note

All claims expressed in this article are solely those of the authors and do not necessarily represent those of their affiliated organizations, or those of the publisher, the editors and the reviewers. Any product that may be evaluated in this article, or claim that may be made by its manufacturer, is not guaranteed or endorsed by the publisher.
